# A Rapid Screening Assay Identifies Monotherapy with Interferon-ß and Combination Therapies with Nucleoside Analogs as Effective Inhibitors of Ebola Virus

**DOI:** 10.1371/journal.pntd.0004364

**Published:** 2016-01-11

**Authors:** Stephen D. S. McCarthy, Beata Majchrzak-Kita, Trina Racine, Hannah N. Kozlowski, Darren P. Baker, Thomas Hoenen, Gary P. Kobinger, Eleanor N. Fish, Donald R. Branch

**Affiliations:** 1 Department of Laboratory Medicine and Pathobiology, University of Toronto, Toronto, Ontario, Canada; 2 Division of Advanced Diagnostics, Infection and Immunity Group, Toronto General Research Institute, Toronto, Ontario, Canada; 3 Special Pathogens Program, National Microbiology Laboratory, Winnipeg, Manitoba, Canada; 4 Biogen, Cambridge, Massachusetts, United States of America; 5 Laboratory of Virology, National Institute of Allergy and Infectious Diseases, National Institutes of Health, Hamilton, Montana, United States of America; 6 Department of Immunology, University of Toronto, Toronto, Ontario, Canada; 7 Centre for Innovation, Canadian Blood Services, Toronto, Ontario, Canada; University of Texas Medical Branch, UNITED STATES

## Abstract

To date there are no approved antiviral drugs for the treatment of Ebola virus disease (EVD). While a number of candidate drugs have shown limited efficacy *in vitro* and/or in non-human primate studies, differences in experimental methodologies make it difficult to compare their therapeutic effectiveness. Using an *in vitro* model of *Ebola Zaire* replication with transcription-competent virus like particles (trVLPs), requiring only level 2 biosafety containment, we compared the activities of the type I interferons (IFNs) IFN-α and IFN-ß, a panel of viral polymerase inhibitors (lamivudine (3TC), zidovudine (AZT) tenofovir (TFV), favipiravir (FPV), the active metabolite of brincidofovir, cidofovir (CDF)), and the estrogen receptor modulator, toremifene (TOR), in inhibiting viral replication in dose-response and time course studies. We also tested 28 two- and 56 three-drug combinations against Ebola replication. IFN-α and IFN-ß inhibited viral replication 24 hours post-infection (IC_50_ 0.038μM and 0.016μM, respectively). 3TC, AZT and TFV inhibited Ebola replication when used alone (50–62%) or in combination (87%). They exhibited lower IC_50_ (0.98–6.2μM) compared with FPV (36.8μM), when administered 24 hours post-infection. Unexpectedly, CDF had a narrow therapeutic window (6.25–25μM). When dosed >50μM, CDF treatment enhanced viral infection. IFN-ß exhibited strong synergy with 3TC (97.3% inhibition) or in triple combination with 3TC and AZT (95.8% inhibition). This study demonstrates that IFNs and viral polymerase inhibitors may have utility in EVD. We identified several 2 and 3 drug combinations with strong anti-Ebola activity, confirmed in studies using fully infectious ZEBOV, providing a rationale for testing combination therapies in animal models of lethal Ebola challenge. These studies open up new possibilities for novel therapeutic options, in particular combination therapies, which could prevent and treat Ebola infection and potentially reduce drug resistance.

## Author Summary

Studies to evaluate the effectiveness of candidate antiviral drugs to inhibit Ebola virus infection have been hampered by the availability and access to level 4 containment facilities. Using a mini-genome model system that generates Ebola virus-like particles that infect cells, we have been able to screen a panel of candidate drugs for antiviral activity, under normal level 2 containment. We compared the activities of 8 different antivirals from 3 drug classes, including drugs repurposed for the treatment of Ebola: type I interferons and nucleoside analogs. Our data indicate that IFN-ß is a potent inhibitor of Ebola virus, contributing to the decision to conduct a clinical trial of IFN-ß treatment for Ebola virus disease in Guinea. Moreover, we identified that 2 and 3 drug combinations inhibit Ebola replication when administered 24 hours post-infection. Drug combinations have important implications for clinical use, since lower doses of each drug are administered, potentially decreasing side-effects and, based on different mechanisms of action, there is less likelihood for the emergence of drug resistance. These studies set the stage for both preclinical and clinical evaluation.

## Introduction

As of December 13, 2015, the current outbreak of Ebola virus disease (EVD) in West Africa has resulted in 28,633 cumulative cases and 11,314 deaths [1]. Two potential vaccine candidates, rVSVΔG-ZEBOV and ChAd3-EBO Z, have shown durable protection from lethal Ebola challenge in mice [2] and macaques [3] respectively, and are part of the phase II/III PREVAIL trial in Liberia and Guinea (https://clinicaltrials.gov/ct2/show/NCT02344407). Other potential therapeutics, such as convalescent plasma and the antibody cocktail ZMapp [4] have been approved for an emergency phase II/III trial in Guinea (https://clinicaltrials.gov/ct2/show/NCT02342171) and a phase I trial in Liberia (https://clinicaltrials.gov/ct2/show/NCT02363322), respectively. However, to date there is no licensed vaccine or treatment for EVD, although improvements in supportive care are increasing survival rates [5].

Repurposing antivirals used for other viral infections, based on knowledge of mechanisms of action, has prompted accumulating interest in the application of different nucleoside/nucleotide analogs and type I interferons (IFNs) for the treatment of Ebola virus disease (EVD). Experimental nucleoside analogs may have therapeutic efficacy for EVD, given the evidence of protection in primate and rodent disease models, 2–6 days after lethal Ebola or the related hemorrhagic Marburg virus challenges [6,7]. Favipiravir, a viral polymerase inhibitor, provides 100% protection when administered 6 days after challenge with a lethal dose of Ebola virus [6] and has been evaluated in the phase II/III JIKI trial in Guinea (https://clinicaltrials.gov/ct2/show/NCT02329054). TKM-100802, a cocktail of siRNAs targeting VP35 and L polymerase and brincidofovir (BCV), a viral polymerase inhibitor that has activity against dsDNA viruses such as adenovirus and cytomegalovirus [8], were also considered for treatment against EVD. The brincidofovir trial was halted, ostensibly because of projections of low recruitment.

Despite infecting different target cells, Ebola and HIV-1 share many similar features early in their replication cycle. Both are RNA viruses that package a viral polymerase (L for Ebola, RT for HIV-1) required for early replication in the cytosol of the host cell [9]. Homology-based structural prediction of the RNA-dependant RNA polymerase of Ebola indicates the polymerase contains conserved structural motifs in the catalytic palm subdomain similar to viral DNA polymerases [10], supportive of nucleoside analogs potentially inhibiting Ebola replication. Inhibiting HIV-1 reverse transcription with nucleoside analogs such as lamivudine (3TC, cytidine analog), zidovudine (AZT, thymidine analog) or tenofovir (TFV, adenosine monophosphate analog) is the basis for highly active antiretroviral treatment (HAART) [11,12]. Nucleoside analogs are on the WHO list of essential medicines and can be deployed in limited resource settings [13]. Moreover, AZT binds RNA through G-C and A-U bases [14], prompting us to evaluate whether these nucleoside analogs might also inhibit Ebola replication.

Type I IFNs mediate diverse biological effects, including cell type-independent antiviral responses and cell type-restricted responses of immunological relevance. IFNs inhibit viral infection by preventing viral entry into target cells and by blocking different stages of the viral replication cycle for different viruses. Moreover, type I IFNs have a critical role in linking the innate and adaptive immune responses to viral challenge. IFN-α/β expression occurs as the earliest non-specific response to viral infection. Indeed, viruses have evolved immune evasion strategies specifically targeted against an IFN response, confirming the importance of IFNs as antivirals. This immune evasion strategy is relevant when one considers the IFN response to Ebola infection [15]. Ebola proteins VP24 and VP35 inhibit host cell systems that lead to IFN production and also inhibit events associated with an IFN response [16–18]. VP24 blocks the binding of importins to phosphorylated STAT1, preventing STAT1 nuclear translocation required for transcription of interferon simulated genes [16]. VP35 binds viral dsRNA, preventing dsRNA degradation [17] and inhibits the phosphorylation of IRF-3 and the SUMOylation of IRF-3 and IRF-7, thereby limiting IFN production [18]. Despite these virally-encoded mechanisms to limit an IFN response to infection, different rodent and non-human primate studies provide evidence for IFN-induced partial protection: the effects of IFN-α/β treatment in lethal Ebola virus infection reduced viremia and prolonged survival [19–21]. Thus, a potential therapeutic effect for IFNs as monotherapy in EVD, or in combination with other anti-Ebola therapies, has not been resolved.

## Results

We employed an established mini-genome system to rapidly evaluate candidate drugs that could inhibit *Ebola Zaire* replication under BSL 2 conditions [22–24]. At the outset we established the experimental conditions for infection with replication and transcription-competent virus like particles (trVLPs), by examining luciferase activity under various transfection and drug treatment conditions, which included transfection with viral support protein plasmids (S1 Fig). We included treatment with maraviroc, a CCR5 inhibitor, that would have no effect on trVLP entry and infection, thereby serving as a negative control for subsequent treatment regimens.

**Fig 1:**
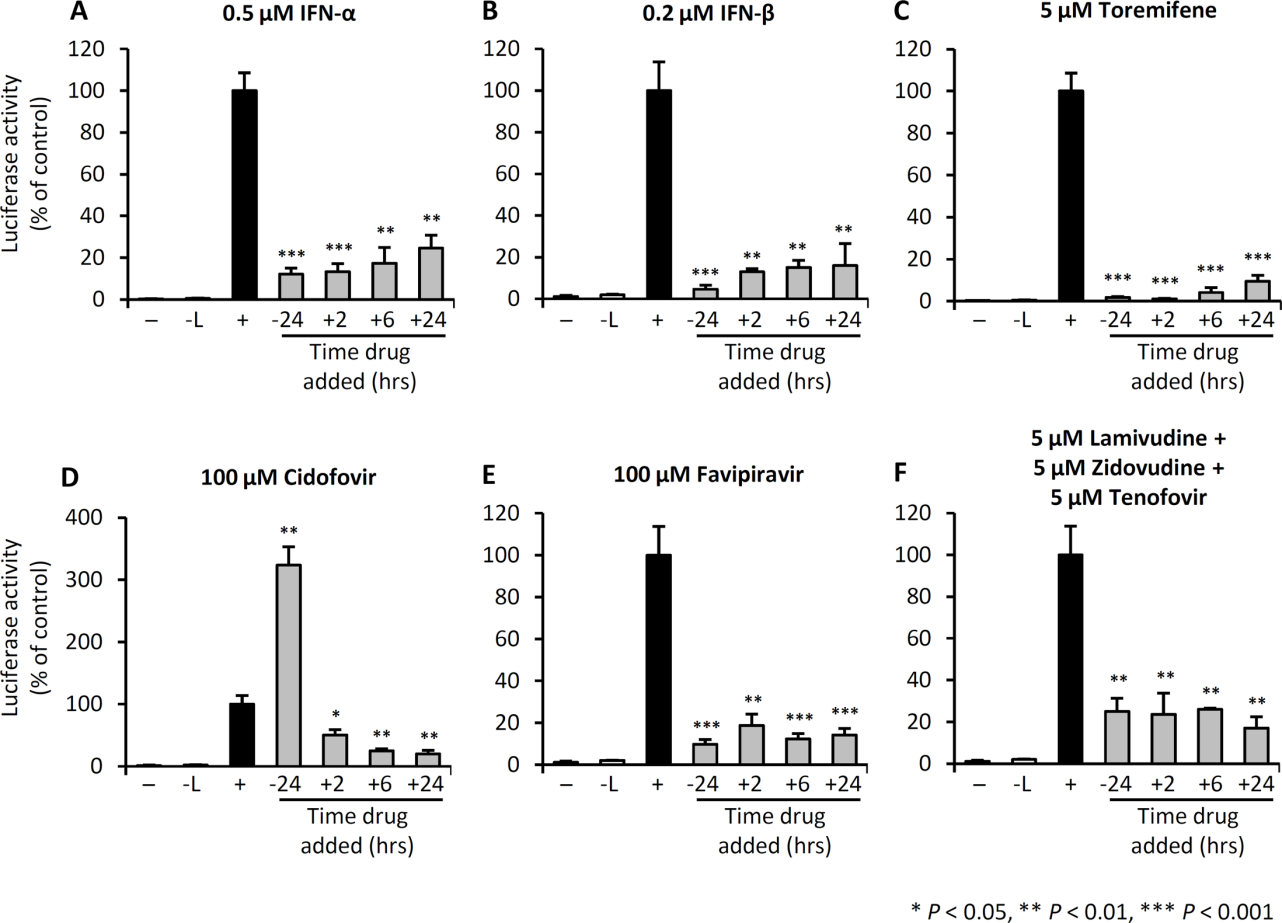
IFNs, toremifene, and nucleoside analogs inhibit trVLP replication. 293 T cells were either left untreated (-), transfected with the mini-genome and the nucleocapsid plasmids NP, VP30, VP35 and T7 (-L), or transfected with the mini-genome and all expression plasmids to permit Ebola mini-genome entry and replication (+). (A-F) Cells were treated with the indicated drugs at the indicated doses, at each of the time points shown. Luciferase activity was measured 4 days post-trVLP infection. Values shown are the means of 4 biological replicates and are representative of 2 independent experiments. Error bars are the standard error of the mean. All drug treatment outcomes were statistically compared with the (+) control group. See also S1 Fig.

In a first series of experiments, we examined the inhibitory effects of IFN-α (0.5μM/10,000 U/mL), IFN-ß (0.2μM/1,000 U/ml), TOR (5μM), CDF (100μM), FPV (100μM), and a combination of 3TC, AZT and TFV (5μM each) on trVLP infection of 293T cells (Fig 1). Specifically, the 293 T cells were treated with the different drugs at four different times relative to infection with trVLP, as indicated. We provide evidence that for each of the individual drugs and for the triple drug combination, at the doses indicated, trVLP infection of 293 T cells is inhibited when treatment is initiated at +2, +6 or +24 hours post-infection. Interestingly, TOR, an estrogen receptor modulator discovered in a high throughput screen as a potent inhibitor of Ebola [25], significantly reduced viral luciferase activity at all time-points tested. For IFN-α, IFN-ß, TOR and FPV treatments, maximal inhibition of trVLP infection was achieved when the cells were treated prior to challenge with trVLP. By contrast, pre-treatment with CDF at 100μM, 24 hours prior to infection with trVLP, resulted in enhanced infection.

**Fig 2:**
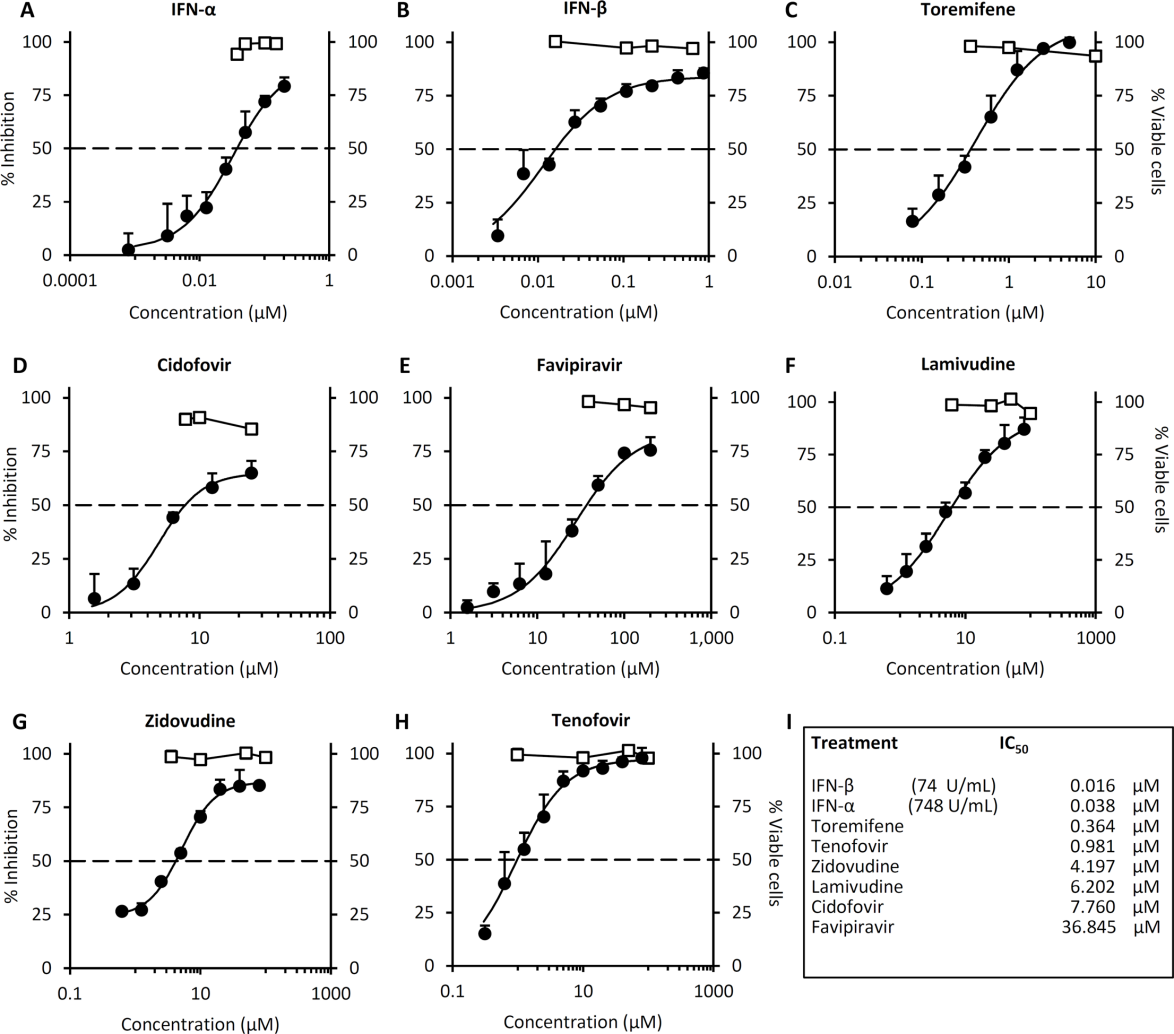
IFNs, toremifene and nucleoside analogs administered 24hrs post-exposure inhibit Ebola-mini-genome replication. 293 T cells were either left untreated (-), transfected with the mini-genome and the nucleocapsid plasmids NP, VP30, VP35 and T7 (-L), or transfected with the mini-genome and all expression plasmids to permit Ebola mini-genome entry and replication (+), as described in Materials and Methods. 24 hours post-trVLP infection, cells were either left untreated, or treated with the indicated drugs (A-I) Dose-response plots for each of the indicated drugs. Luciferase activity (black circles) or cell viability (white squares) was measured 4 days post-infection (3 days after drug treatment). Values are the means of 4 biological replicates and are representative of 2 independent experiments. Error bars are the standard error of the mean. See also S2 and S3 Figs.

In subsequent dose-response studies, we compared the inhibitory effects of IFN-α, IFN-ß, TOR, CDF, FPV, 3TC, AZT or TFV when administered 24 hours post trVLP infection (Fig 2). The data in Fig 2I summarize the IC_50_ dose for each drug. The IFNs exhibited the lowest IC_50_ values at 0.016μM for IFN-ß and 0.038μM for IFN-α. The data show a log-fold difference in IC_50_ values for IFN-α and IFN-ß when compared in terms of U/ml, the norm for antiviral activity measurements (Fig 2A and 2B). TOR had the next lowest IC_50_ (0.36μM) and completely inhibited infection at doses > 5μM (Fig 2C). TFV had an IC_50_ at 0.98μM. CDF, 3TC and AZT all exhibited similar IC_50_ values in the dose range 4.2–7.8μM, while FPV had the highest IC_50_ of the nucleoside analogs at 36.8μM. At their IC_50_ concentration, none of these drugs directly inhibited luciferase reporter activity (S2 Fig). We observed a relatively small antiviral dose range for CDF (1.5–25μM) (Fig 2D), beyond which the drug appeared to enhance viral infection (S3 Fig). In cell viability assays we observe that at doses >10μM CDF affect cell viability, confounding the interpretation of the effects of CDF on viral replication.

**Fig 3:**
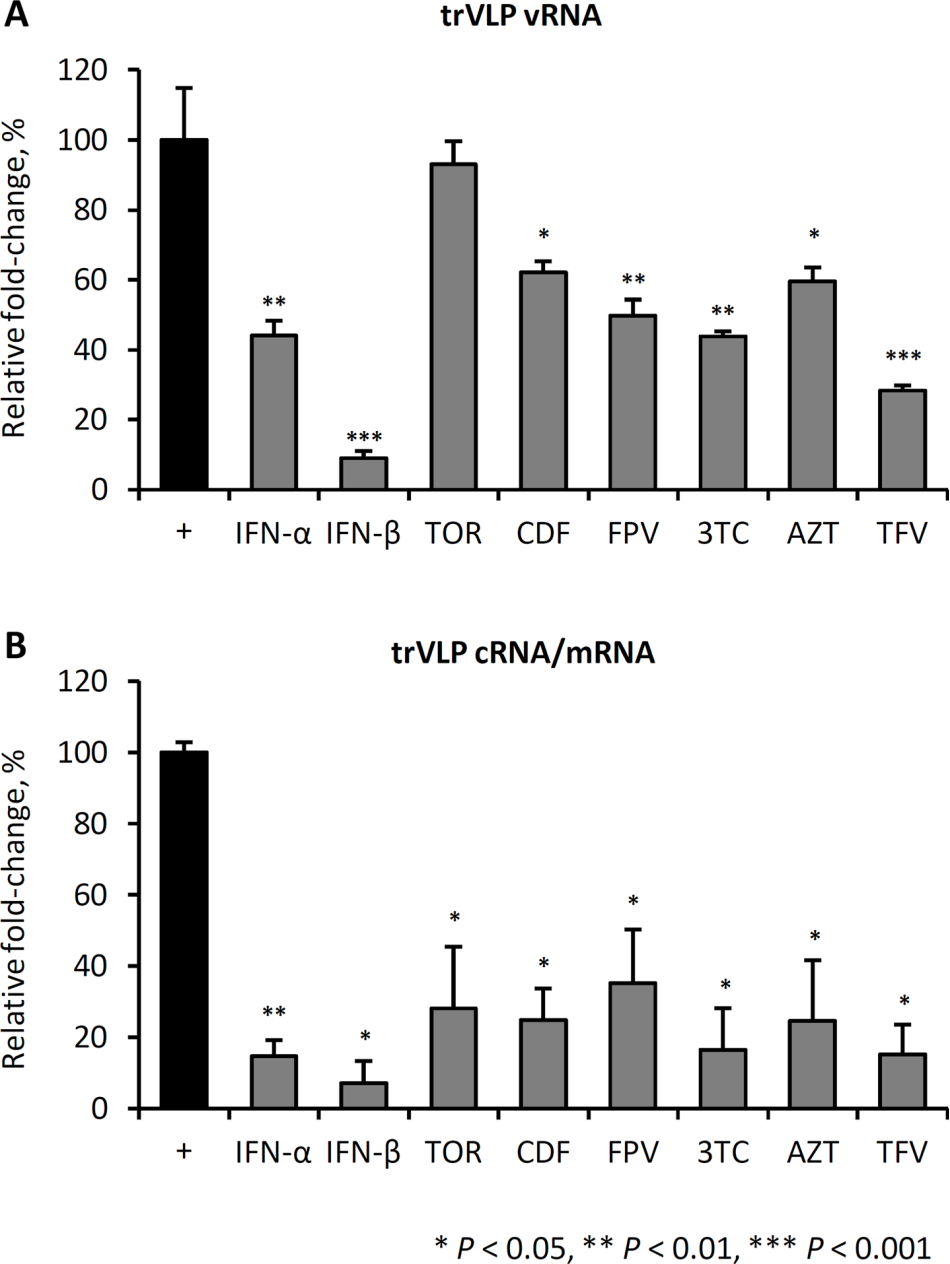
IFNs, toremifene and nucleoside analogs reduce trVLP replication and transcription. 293 T cells were transfected with support plasmids and infected with trVLP. 24 hours post-infection, cells were treated with the indicated drugs at their IC_50_ doses, determined from Fig 2I. At 48hrs post-infection, total RNA was extracted, reverse transcribed, then quantified by qPCR. Relative fold-change in -ve sense vRNA transcripts (A) and +ve sense cRNA and mRNA (B) was compared with infected, untreated cells (solvent,+ control). Technical duplicates were examined by qPCR, and means are the average of three biological replicates. Error bars are the standard error around the mean.

In an orthogonal assay to confirm these findings, we next measured viral replication and transcription by qRT-PCR, following trVLP infection. trVLP-infected cells were either left untreated, or treated with the different drugs 24 hours post-infection, then viral replication and transcription evaluated 24 hours later (Fig 3). All treatments, with the exception of TOR, significantly reduced the amount of genomic vRNA detected within cells (Fig 3A) and all treatments significantly reduced the synthesis of cRNA and mRNA isolated from infected cells (Fig 3B). Notably, IFN-ß treatment of trVLP-infected cells resulted in the greatest reduction in viral replication and transcription.

**Fig 4:**
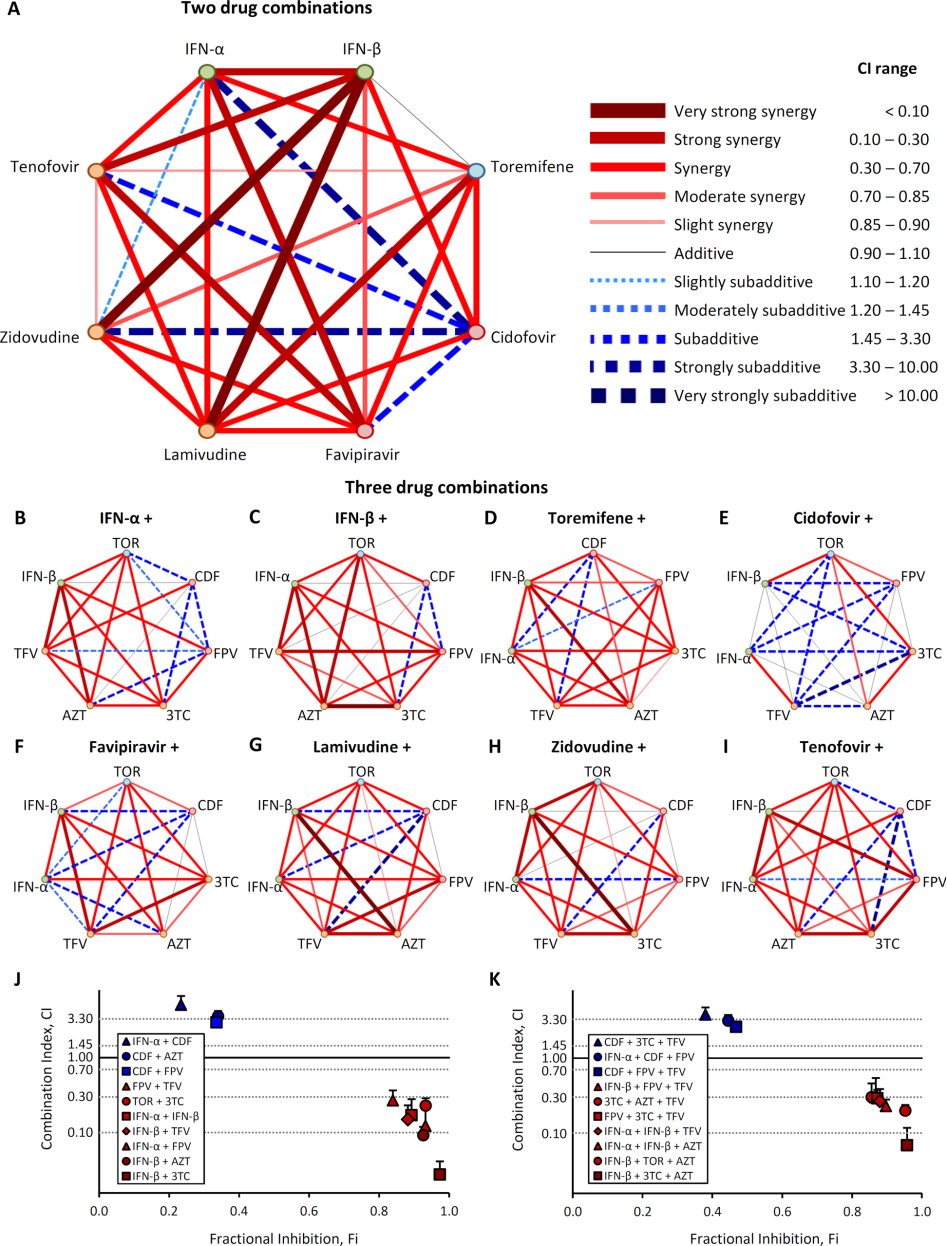
2 and 3 drug combinations synergistically inhibit Ebola trVLP infection. 293 T cells were either left untreated, transfected with the mini-genome and the support plasmids NP, VP30, VP35, T7 but not L, or the mini-genome plus all the support plasmids. (A) Polygonogram of 28 two-drug combinations (at monotherapy IC_50_ doses) administered at 24 hrs post-trVLP infection, then luciferase activity evaluated 3 days later. A thick red line represents strong synergy between two drugs (CI<1), a thin black line represents additive effects (CI = 1), and a thick blue line represents strong sub-additive (less than additive) between two drugs (CI>1). (B-I) Polygonograms of 56 three-drug combinations. The backbone drug in each triple combination is listed above the heptagon, and the synergism/sub-additive effect of the additional two drugs is represented within each heptagon. (J-K) Combination index (CI) vs. fractional inhibition (Fi) plots of the most synergistic and sub-additive double (J) and triple (K) drug combinations on trVLP luciferase activity. Dotted lines identify thresholds of synergy/additive effects. Values shown are the means of 2–4 biological replicates in 2 independent experiments. Error bars are the standard error of the mean. See also S1 and S2 Tables.

Next we examined the effectiveness of two and three drug combinations on trVLP infection. We first examined 28 two-drug combinations, using each drug at its IC_50_ value, and used the median-effect equation and combination index theorem [26] to determine drug synergy, additive or sub-additive effects (Fig 4A). Synergy is defined as greater than additive effect when drugs were combined (CI<1), additive as the effect expected when combining each drug (CI = 1) and sub-additive as a smaller than expected additive effect (CI>1). When administered 24 hours post-infection, many of the two-drug combinations showed strong synergism in inhibiting trVLP replication (Fig 4J), with IFN-β + 3TC demonstrating the greatest synergism (97.3% inhibition, CI = 0.028). 3TC was synergistic with all seven other drugs tested. Notably, when CDF was used in combination with FPV, AZT, TFV or IFN-α, it produced a sub-additive effect.

Next we tested all possible 56 three-drug combinations, using each drug at its IC_50_ value, to assess whether adding a third drug enhanced efficacy compared with two-drug combinations (Fig 4B–4I). This series of experiments served to validate our two-drug findings, as synergistic two-drug combinations such as IFN-β + 3TC and IFN-β + AZT, predicted strong synergy for the triple drug combination of IFN-ß + 3TC + AZT. As anticipated from the two-drug polygonogram, CDF was sub-additive when combined in three-drug combinations (Fig 4E). This was most evident even when CDF was administered in conjunction with two-drug combinations that had shown strong synergy, such as IFN-β + 3TC or FPV + TFV, further indicating that CDF diminishes the antiviral effects of other drugs. IFN-ß, 3TC, AZT and TFV all promoted strong synergism when included in triple drug combinations, with IFN-β + AZT specifically providing strong synergism when combined in three unique triple therapies.

From these two-drug and three-drug screens, we calculated the combination index (CI) and fractional inhibition (Fi) (Fig 4J and 4K). Many of the synergistic drug combinations (i.e. low CI) included one nucleoside analog and an IFN, while those drug combinations that were sub-additive all included CDF. IFN-β was predominant in the most efficacious two- and three-drug combinations. In particular, IFN-β + 3TC and IFN-β + 3TC + AZT consistently exhibited the strongest synergism and highest Fi when administered 24 hours post-infection. Refer also to S1 and S2 Tables.

**Fig 5:**
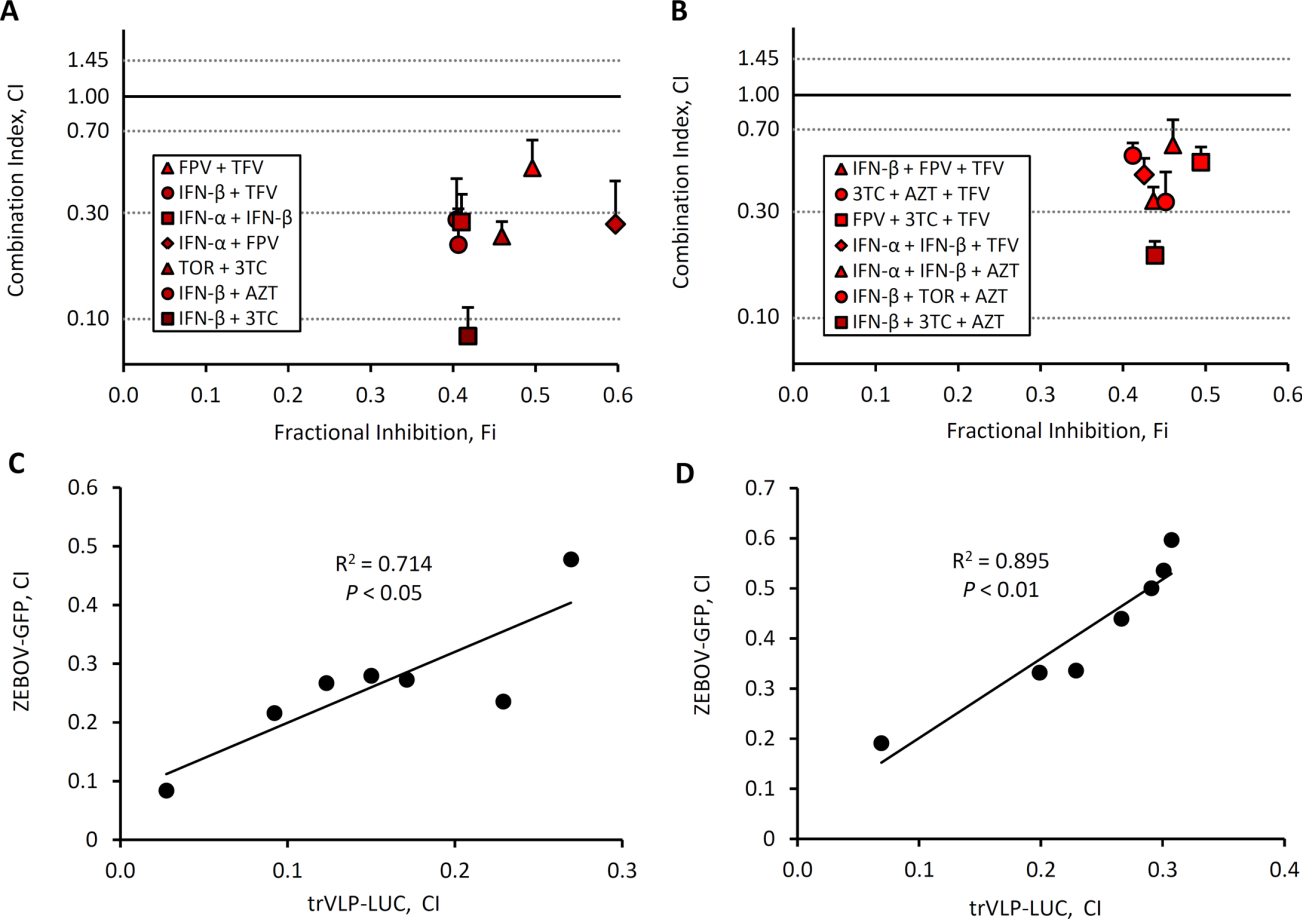
Synergistic 2 and 3 drug combinations against trVLP-LUC infection inhibit fully infectious ZEBOV-GFP. (A-B) 293 T cells were infected with ZEBOV-eGFP at an MOI of 0.1, then treated with 2 and 3 drug combinations as indicated, 24 hours post-infection, at their monotherapy IC_25_ doses. GFP fluorescence was measured 3 days post-infection. Values are the means of 4 biological replicates, and error bars represent the standard error of the mean. Data are representative of 2 independent experiments. The combination index (CI) was plotted against the fractional inhibition (Fi) for each drug combination. (C-D) Plot of CIs for ZEBOV infections compared with CIs for trVLP infections.

In a final series of experiments, in order to validate our findings from the trVLP infection studies, we examined the antiviral effectiveness of IFN-ß, IFN-α, TOR, FVP, AZT, 3TC and TFV in 293T cells infected with ZEBOV (ZEBOV contained an eGFP reporter). CDF was excluded from these experiments. Initial dose-response studies were conducted at doses reflective of those used in the trVLP experiments in Fig 2. A higher dose of each drug was required to inhibit ZEBOV infection compared with trVLP infection (S4 Fig). Using the IC_25_ of each drug, we next evaluated 2 and 3 drug combinations for additive or synergistic effects against ZEBOV infection. All seven 2 drug combinations were synergistic (low CI) (Fig 5A), similar to the most synergistic combinations against trVLP in Fig 4J. IFN-β + 3TC proved to be the most synergistic 2 drug combination, analogous to trVLP infection. Of the most synergistic 3 drug combinations identified in the trVLP infection system, all seven exhibited synergy against ZEBOV infection, with IFN-β + 3TC + AZT and IFN-β + TOR + AZT exhibiting the strongest synergy (Fig 5B). The CIs determined from trVLP infection correlated well with those determined using ZEBOV infection; specifically, the correlation coefficients (R^2^ values) confirm this (Fig 5C and 5D).

## Discussion

In September 2014, the WHO hosted a conference to facilitate development of a global action plan to deal with the Ebola outbreak in West Africa. Delegates from affected West African countries, ethicists, scientists, health care providers, logisticians and representatives from different funding agencies were in attendance. A committee had been struck to evaluate the different vaccine candidates and therapeutic interventions being developed, which subsequently received an overwhelming number of submissions for consideration, and was hampered by an inability to compare antiviral effectiveness, since *in vitro* and pre-clinical *in vivo* model systems vary, treatment regimens vary from prophylaxis to post-exposure administration, and direct readouts of antiviral efficacy differ. Moreover, given the virulence and high mortality associated with EVD, all of these studies have been conducted under BSL 4 conditions, limiting the number of laboratories that can engage in these antiviral studies. Cognizant of these limitations, we employed the trVLP model system to compare the antiviral effectiveness of eight antiviral candidates from three drug classes. We evaluated their antiviral activities in the context of inhibition of Ebola replication, using this mini-genome model that allows for rapid comparisons among compounds under BSL 2 conditions. The tetracistronic minigenome represents the most sophisticated *in vitro* replication model of Ebola virus to date. trVLPs proceed through every replication step as wild-type Ebola virus, and have been tested in multiple cell lines. Using TOR, there has been some validation of the trVLP assay. Specifically, TOR has been evaluated in limiting Ebola virus infection of VeroE6 and HepG2 cells, and exhibited IC_50_ values of 0.2 μM and 0.03 μM, respectively [27], in line with the IC_50_ dose for TOR (0.36μM) observed with trVLP infection. Likewise, the IC_50_ identified in the trVLP system for FPV (36.8μM), is consistent with that of 67μM recorded using Ebola virus infection [6], suggesting that this Ebola mini-genome system has relevance for screening potential antiviral compounds. Indeed, our validation studies using ZEBOV (ZEBOV-eGFP) suggest that the trVLP infection model has utility as an *in vitro* screening assay when comparing different drugs as monotherapies or in 2 and 3 drug combinations.

As mentioned, the Ebola virus encodes in its genome factors that limit a type I IFN response to infection [16–18]. Yet, both rodent and non-human primate studies suggest that IFN-α and IFN-ß treatment can confer partial protection from infection, reducing viremia and prolonging survival [19–21], suggesting that it may be possible to override the inhibitory effects of the virus by treatment with IFN. At the outset, we conducted a series of experiments to compare the antiviral activities of IFN-α and IFN-ß in the trVLP infection system, and our findings suggest that whether treatment is administered prior to or post-infection, both IFN-α and IFN-ß exhibit antiviral activity. These findings only have relevance for the *direct* antiviral activities of these IFNs, since the effects of IFN-α or IFN-ß on immune modulation for viral clearance cannot be determined using this system. Nevertheless, these data contributed to the decision to conduct a clinical trial of IFN-ß treatment for EVD in Guinea.

We provide evidence that the nucleoside/nucleotide analogs 3TC, AZT, TFV, FPV and CDF inhibit Ebola trVLP replication *in vitro*. The results with 3TC are in contrast to published data that show no evidence for 3TC inhibiting Ebola virus infection *in vitro* [27]. These studies examined the antiviral effectiveness of 3TC when administered one hour prior to infection, in contrast to our studies that have focused on post-exposure protection. In cells, the kinetics of 3TC phosphorylation are such that a minimum of four hours are required for optimal activity, perhaps distinguishing why our 24 hour pre-treatment, specifically a combination treatment, offered protection. Post-exposure treatment with 3TC and the other nucleoside/nucleotide analogs we examined, would more likely reveal activity against viral RNA synthesis than pre-treatment. When comparing the IC_50_ values of each of the nucleoside analogs that we tested, TFV exhibited the lowest IC_50_ at ~1μM. Whether this reflects the fact that this adenosine monophosphate analog only requires two phosphorylation events to become an active drug versus three for the other nucleoside analogs, remains undetermined. Extensive published data reveal both the safety profiles [11,28,29] and the biodistribution of 3TC, AZT and TFV in the circulation and liver [30,31], the same compartments where Ebola infects monocytes, macrophages, dendritic cells, endothelial cells and hepatocytes. Moreover, drug interactions with other nucleoside analogs have been well studied: e.g. tenofovir disoproxil fumarate, when used alone or in combination with emtricitabine effectively prevents HIV-1 infection in antiretroviral pre-exposure prophylaxis (PrEP) [29].

Our studies also revealed that the active metabolite of brincidofovir, CDF, has a narrow therapeutic window of efficacy (6.25–25μM) when assessed in the trVLP assay, enhancing viral replication at higher doses when added either prior to or post-infection. In cell viability assays, CDF exhibits cytotoxicity at doses >10μM. These findings suggest that caution is required if CDF is to be considered further for the treatment of EVD, specifically that phase I/II trials define the safety profile of this drug for EVD.

Another advantage of this *in vitro* system is that it allowed us to evaluate various 2 and 3 drug combinations and demonstrates that combination treatments limit viral replication up to 97.3%. A benefit of combination treatment is the potential to limit/avoid the emergence of drug resistance. Interestingly, IFN-ß was predominant among all the 8 antivirals considered in terms of contributing very strong synergism in combination treatments: e.g. IFN-ß + 3TC; IFN-ß + 3TC + AZT. Using this system, we observe that FPV, when administered 24 hours post-infection, has an IC_50_ of ~ 37μM. To date, the phase II/III JIKI trial examining the efficacy of FPV against EVD has reported only modestly encouraging results. In our 2 drug combination treatment studies we show that, with the exception of CDF, whenever FPV is included, synergy occurs, effectively reducing the CI. It may transpire that for treating EVD, FPV is most effective in a drug combination regimen.

Viewed altogether, we present an *in vitro* Ebola trVLP screening system, that requires only level 2 biocontainment, which allowed us to compare the antiviral activities of 8 compounds, either alone or in combination. We provide evidence that IFNs are effective inhibitors of Ebola replication, with IFN-ß exhibiting greater efficacy over IFN-α, or when used in combination with nucleoside analogs. We infer from our data that whether IFN-ß treatment is administered 24 hours prior to, or up to 24 hours post-infection, reduced Ebola replication is achieved. As additional antiviral therapeutic candidates become available, we now have the capability to measure and compare their direct antiviral activities with the existing panel. This allows for rapid *in vitro* evaluation and the opportunity to prioritize antiviral candidates for further pre-clinical and clinical trial studies.

## Materials and Methods

### Cell culture and trVLP infection

We employed an established mini-genome system to rapidly evaluate candidate drugs that could inhibit *Ebola Zaire* replication under BSL 2 conditions [22]. The mini-genome encodes 3 of the 7 Ebola proteins (VP24, VP40 and GP_1,2_) and a luciferase reporter gene. Expression plasmids for the remaining four Ebola nucleocapsid proteins (L, NP, VP30 and VP35) were also included during transfection. Cell culture conditions and virus infections were performed as previously described [22]. Briefly, 80,000 producer 293 T cells (American Type Culture Collection; ATCC, Rockville, USA) were seeded in individual wells of 24-well plates in 400μL Dulbecco’s Modified Eagle Medium (DMEM) containing 10% FBS, 1% penicillin and 1% streptomycin, and grown in 5% C0_2_ atmosphere at 37°C. Cells were transfected with the viral replication protein plasmids (L, NP, VP30, VP35), a tetracistronic Ebola mini-genome and the T7 polymerase, using the CalPhos Mammalian Transfection Kit (Clontech Laboratories). 24 hours later, medium was replaced with 800μL DMEM with 5% FBS. The replication and transcription-competent virus like particles (trVLPs) were harvested 3 days later. Virus stock was frozen at -80°C.

For infection, 293 T target cells were seeded at 80,000 cells in 400μL of DMEM supplemented with 10% FBS. Target cells were then transfected with the four viral replication protein plasmids, as well as Tim-1, to allow efficient virus binding and entry. 24hr post-transfection, 25μL of trVLP stock was diluted in 600μL of DMEM with 5% FBS, warmed to 37°C for 30 min, then added to target cells. Medium was removed the following day and replaced with 800μL DMEM with 5% FBS. Four days post-infection, the medium was aspirated and cells were re-suspended in 200μL of 1x *Renila* Luciferase Assay Lysis Buffer (*Renilla* Luciferase Assay System, Promega). Lysates were assayed for luciferase activity.

### ZEBOV-eGFP infection

We generated recombinant ZEBOV expressing enhanced green fluorescent protein (eGFP) from cDNA clones of full-length infectious ZEBOV, as previously described [32]. The eGFP reporter protein was expressed as an eighth gene, and the virus exhibited an *in vitro* phenotype similar to wild-type ZEBOV. Notably, *in vivo*, incorporation of GFP into wild-type ZEBOV results in some attenuation of disease [32]. All work with infectious ZEBOV was performed in biosafety level 4 (BSL4), at the National Microbiology Laboratory of the Public Health Agency of Canada in Winnipeg, Manitoba.

30,000 293 T cells were seeded in 96-well plates in 100μL DMEM with 10% FBS. 24 hours thereafter, the medium was replaced with 100μL DMEM with 10% FBS containing ZEBOV-GFP at an MOI of 0.1. 24 hours post-infection, the medium was removed and replaced with 200μL of DMEM with 5% FBS, or 190μL DMEM with 5% FBS and 10μL of single or combinations of drugs. eGFP fluorescence was measured 3 days post-infection using a Synergy HTX Multi-Mode Microplate Reader (BioTek).

### Drugs

For these experiments, we used toremifiene citrate (TOR; Sigma), cidofovir hydrate (CDF; Sigma) favipiravir (FPV, T-705; Cellagen Technology), lamivudine (3TC; Sigma) zidovudine (AZT), tenofovir (TFV) maraviroc (MVC; NIH AIDS Reagent Program), Infergen (IFN alfacon-1, Pharmunion Bsv Development Ltd.) or human interferon beta-1a (IFN-β, Avonex; Biogen).

### Mini-genome RNA extraction and qRT-PCR quantification of viral RNA

Forty-eight hours after trVLP infection, medium was aspirated from 293 T cells that had either been left untreated or treated with the various drugs and total RNA extracted from cell lysates with 500μL of TRIzol (Thermo Fisher Scientific). cDNA synthesis was performed on 5 μg of total RNA, using the First-Strand cDNA Synthesis Kit (GE Healthcare Life Sciences), according to the manufacturer’s instructions. A 20 μl reaction also contained bulk first-strand cDNA reaction mix, DTT solution and 40 pmol of one of two trVLP specific primers [33]: vRNA forward (5’-GGC CTC TTC TTA TTT ATG GCG A -3’), or cRNA/mRNA reverse (5’-AGA ACC ATT ACC AGA TTT GCC TGA-3’). Both primers were synthesized by the Center for Applied Genomics (The Hospital for Sick Children, Toronto, Canada). Real-time qPCR reactions (25 μl) were conducted in duplicate, using the Rotor-Gene RG-3000 thermocycler (Corbett Research, Montreal, Canada). Each reaction contained 100 ng template cDNA, 12.5 μL 2 x SYBR Green PCR Master Mix (Applied Biosystems, Warrington, UK), 300 nM of both the forward (vRNA) and reverse (cRNA/mRNA) primers, and PCR grade H_2_O (Roche Diagnostics, Indianapolis, USA). Samples lacking reverse transcriptase (No RT) during first-strand cDNA synthesis served as negative controls. Cycling parameters were as follows: initial denaturation at 95°C for 10 min, followed by 40 cycles of amplification with 95°C for 15 seconds, 56°C for 30 seconds, and 60°C for 30 seconds. Biological triplicates in the drug-treated groups were normalized to the average Ct of infected cells given DMSO solvent alone, by the 2^-ΔCT^ comparative C_T_ method.

### Cell viability assay

Dose-response cytotoxicity/viability assays were conducted in 293 T cells 4 days post-infection for each of the drugs examined, either alone or in the various combinations indicated, using the MTT assay as previously described [34].

### Statistics

Means were compared using a two-tailed, unpaired Student’s *t* test and corrected for multiple comparisons. For all figures, (*) denotes a *p* value <0.05, (**) denotes a *p* value <0.01 and (***) denotes a *p* value <0.001. Error bars shown are the standard error around the mean (SEM). Synergy between two and three-drug combinations, combination index (CI) and dose-reduction index (DRI) were calculated with CompuSyn Version 1.0 [26]. The coefficient of determination (R^2^) was determined for simple linear regressions.

## Supporting Information

S1 Fig IFNs and nucleoside analogs inhibit Ebola mini-genome replication *in vitro*. (A) 293 T cells were either left untreated (-), transfected with the mini-genome and the viral support protein plasmids NP, VP30, VP35, T7 but not L (-L), or transfected with the mini-genome and all support plasmids that permit Ebola mini-genome entry and replication (+). At -24, 0, and +24 hours relative to infection, cells were treated with 5μM of maraviroc (MVC), lamivudine (3TC), zidovudine (AZT), or tenofovir (TFV), or combinations of the three nucleoside analogs. Luciferase activity was measured four days after infection with trVLPs. (B) Luciferase activity of first passage of 293 T cells (1) treated with nucleoside analogs, and second passage of 293 T cells (2) treated with nucleoside analogs. (C) Luciferase activity of 293 T cells after treating with 5μM combination of nucleoside analogs, cidofovir (CDF) or favipiravir (FPV). (D) Luciferase activity of 293 T cells treated with IFN-α or IFN-β. The values are means of four biological replicates and are representative of two independent experiments. Error bars are the standard error of the mean. All drug treatment outcomes were statistically compared with the (+) control group in panels A, C and D.
(TIF)

S2 Fig No direct effect of drugs on Renilla luciferase reporter assay. 293 T cells transfected and then infected with trVLP were lysed 4 days post-infection. Cell lysate suspended in CCLR reagent was aliquoted into separate tubes and spiked with each drug at the IC_50_ dose from Fig 2I or DMSO solvent (+). Luciferase activity was then quantified. Values are the means of 3 independent experiments. Error bars are the standard error of the mean.
(TIF)

S3 Fig CDF treatment increases trVLP replication. 293 T cells were transfected with all expression plasmids that permit Ebola mini-genome entry and replication, then treated with the indicated doses of CDF 24hrs post-trVLP infection. Luciferase activity was measured four days after cells were infected (3 days post-CDF treatment). Values are the means of 4 biological replicates and are representative of 2 independent experiments. Error bars are the standard error of the mean. CDF treatment outcomes were compared with the (+) control group.
(TIF)

S4 Fig IFNs, toremifene and nucleoside analogs administered 24hrs post-exposure inhibit ZEBOV-GFP. (A-H) 293 T cells were infected with ZEBOV-GFP (MOI = 0.1). Twenty-four hours post-infection cells were either left untreated, or treated with the indicated drugs at the indicated doses. Intracellular GFP was measured 48 hours later and the percent inhibition quantified relative to infected, untreated cells (DMSO solvent control). Values are the means of 4 biological replicates. Error bars are the standard error of the mean.
(TIF)

S1 Table Fi and CI values for two-drug combination treatments. Experimental details are as described in the legend to Fig 4. The fractional inhibition, combination index and strength of synergy (+), additive effects (+/-) or sub-additive (-) of each 2 drug combination therapy are shown.
(TIF)

S2 Table Fi and CI values for three-drug combination treatments. Experimental details are as described in the legend to Fig 4. The fractional inhibition, combination index and strength of synergy (+), additive effects (+/-) or sub-additive (-) of each 3 drug combination therapy are shown.
(TIF)
