# Supplementary figures and images for: A Rapid Screening Assay Identifies Monotherapy with Interferon-ß and Combination Therapies with Nucleoside Analogs as Effective Inhibitors of Ebola Virus
Source: PLoS Negl Trop Dis. 2016 Jan 11;10(1):e0004364. doi: 10.1371/journal.pntd.0004364 (PMC4709101; doi:10.1371/journal.pntd.0004364)

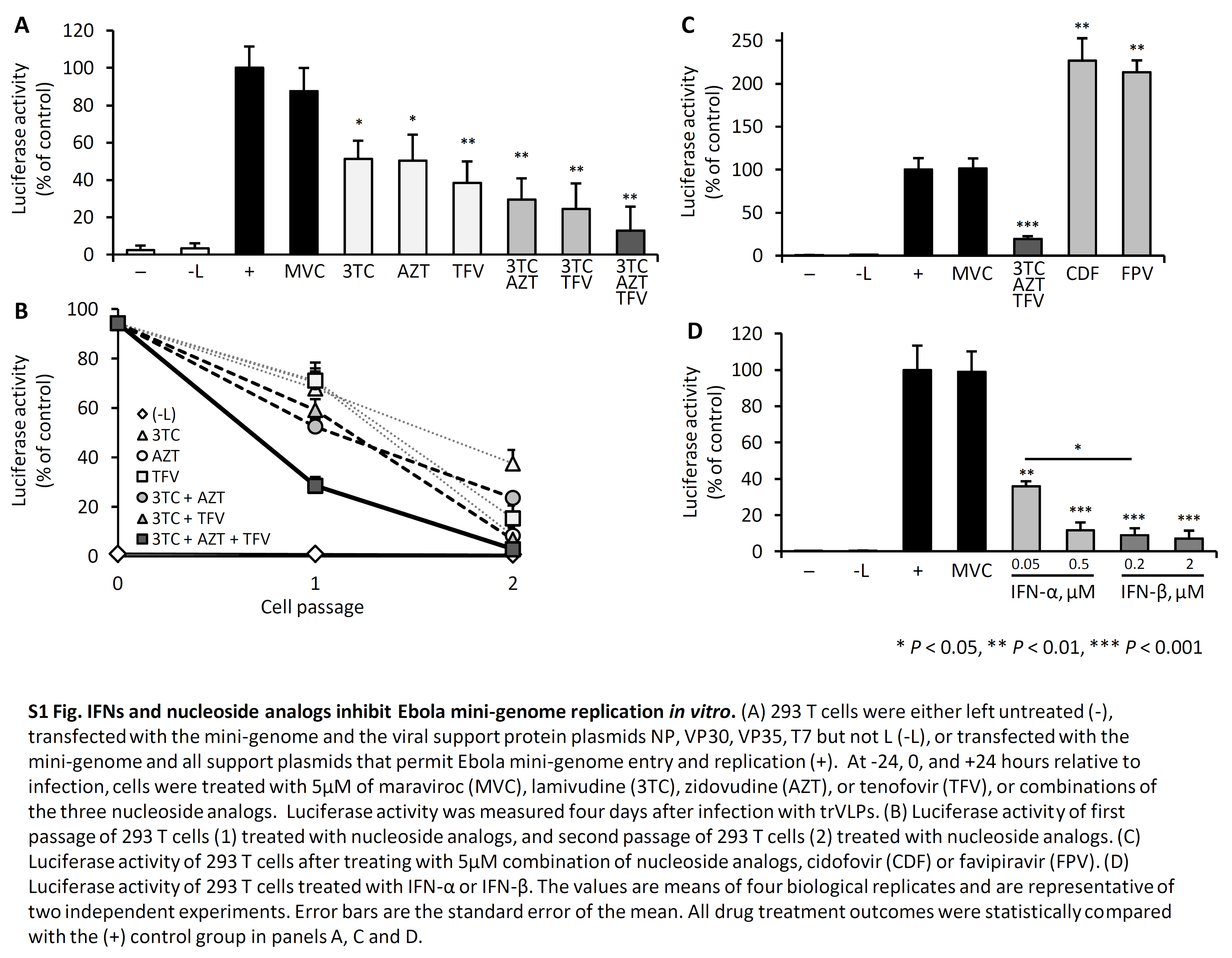

Supplement: S1 Fig — (A) 293 T cells were either left untreated (-), transfected with the mini-genome and the viral support protein plasmids NP, VP30, VP35, T7 but not L (-L), or transfected with the mini-genome and all support plasmids that permit Ebola mini-genome entry and replication (+). At -24, 0, and +24 hours relative to infection, cells were treated with 5μM of maraviroc (MVC), lamivudine (3TC), zidovudine (AZT), or tenofovir (TFV), or combinations of the three nucleoside analogs. Luciferase activity was measured four days after infection with trVLPs. (B) Luciferase activity of first passage of 293 T cells (1) treated with nucleoside analogs, and second passage of 293 T cells (2) treated with nucleoside analogs. (C) Luciferase activity of 293 T cells after treating with 5μM combination of nucleoside analogs, cidofovir (CDF) or favipiravir (FPV). (D) Luciferase activity of 293 T cells treated with IFN-α or IFN-β. The values are means of four biological replicates and are representative of two independent experiments. Error bars are the standard error of the mean. All drug treatment outcomes were statistically compared with the (+) control group in panels A, C and D. (TIF) [file pntd.0004364.s001.tif]

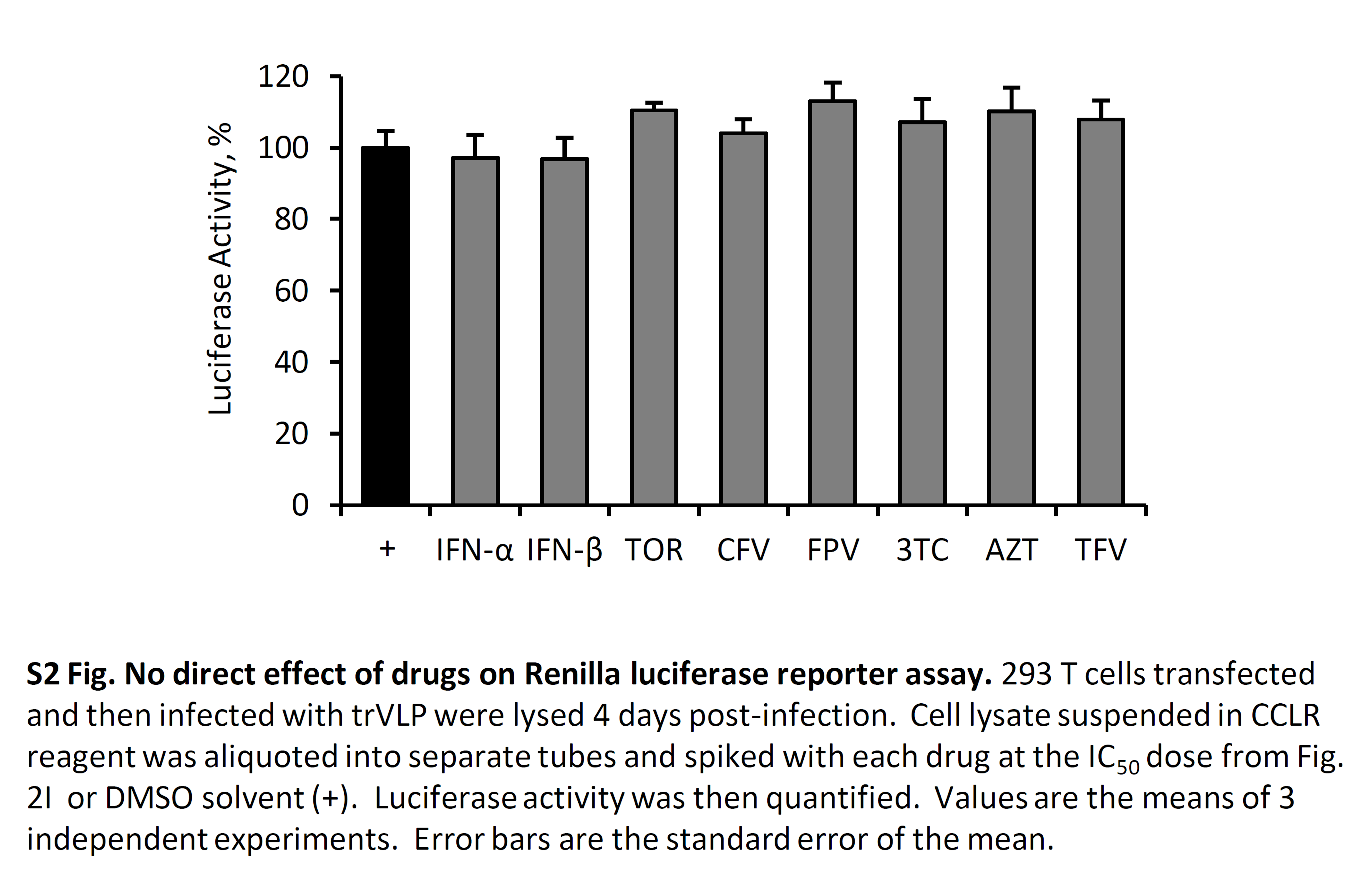

Supplement: S2 Fig — 293 T cells transfected and then infected with trVLP were lysed 4 days post-infection. Cell lysate suspended in CCLR reagent was aliquoted into separate tubes and spiked with each drug at the IC50 dose from Fig 2I or DMSO solvent (+). Luciferase activity was then quantified. Values are the means of 3 independent experiments. Error bars are the standard error of the mean. (TIF) [file pntd.0004364.s002.tif]

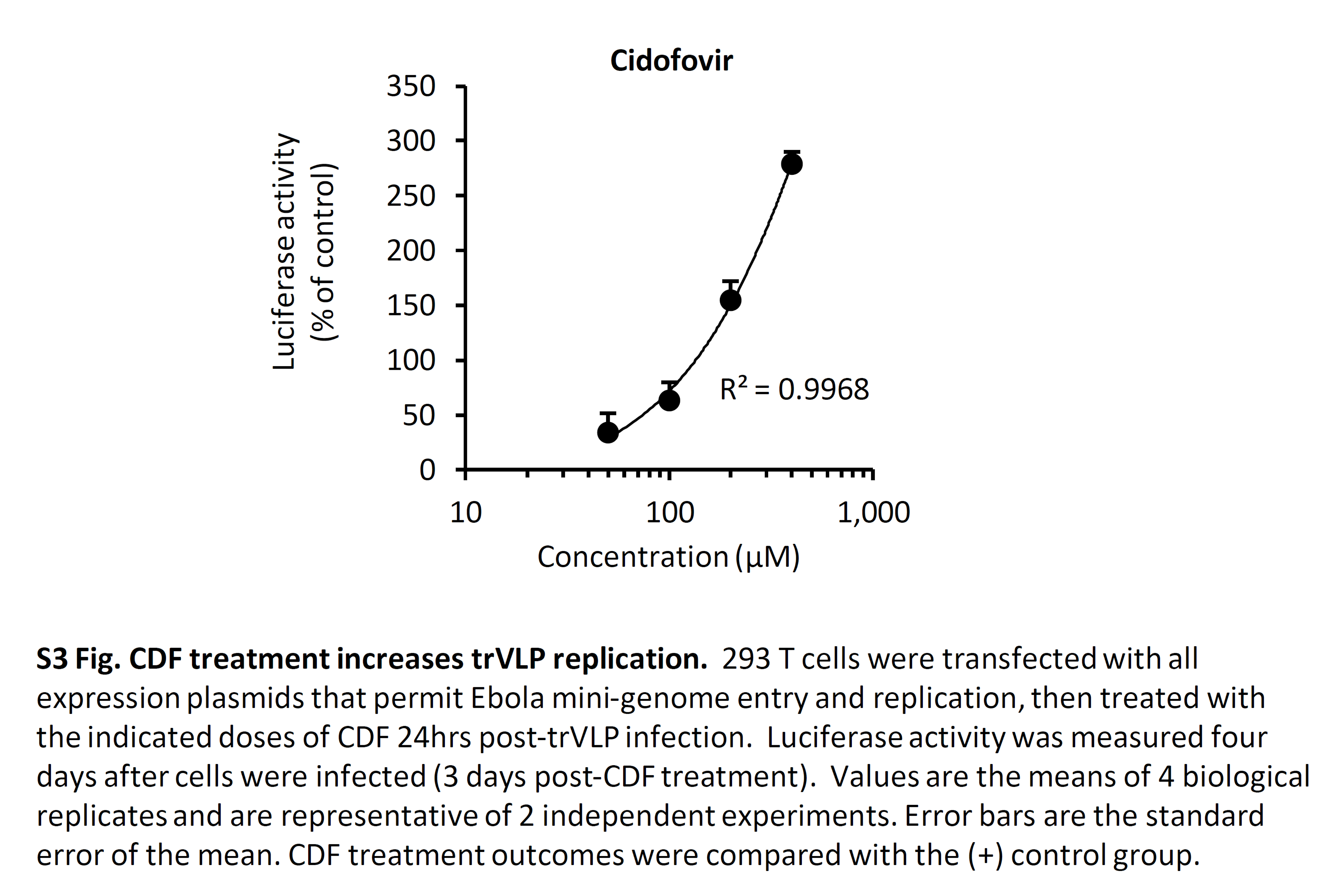

Supplement: S3 Fig — 293 T cells were transfected with all expression plasmids that permit Ebola mini-genome entry and replication, then treated with the indicated doses of CDF 24hrs post-trVLP infection. Luciferase activity was measured four days after cells were infected (3 days post-CDF treatment). Values are the means of 4 biological replicates and are representative of 2 independent experiments. Error bars are the standard error of the mean. CDF treatment outcomes were compared with the (+) control group. (TIF) [file pntd.0004364.s003.tif]

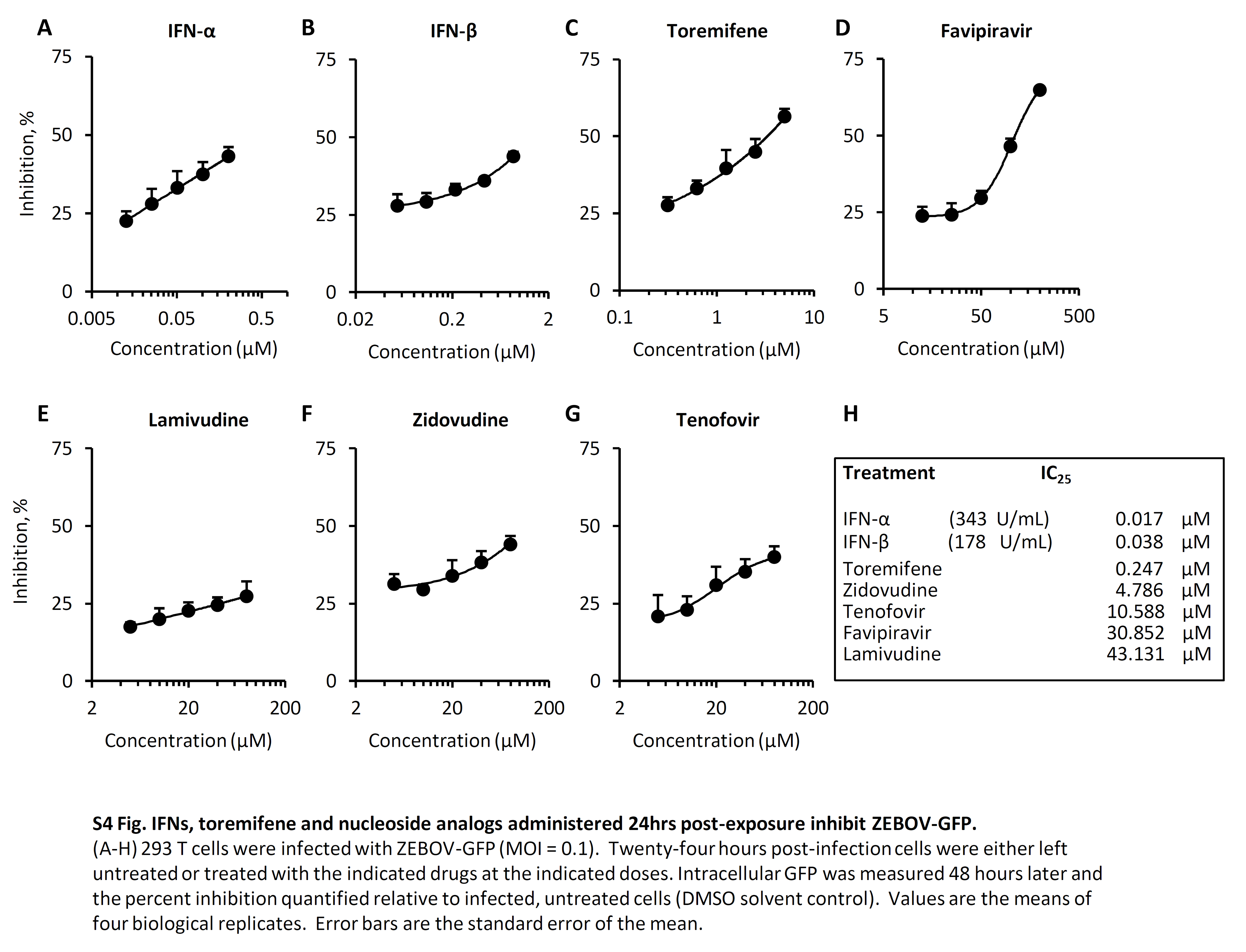

Supplement: S4 Fig — (A-H) 293 T cells were infected with ZEBOV-GFP (MOI = 0.1). Twenty-four hours post-infection cells were either left untreated, or treated with the indicated drugs at the indicated doses. Intracellular GFP was measured 48 hours later and the percent inhibition quantified relative to infected, untreated cells (DMSO solvent control). Values are the means of 4 biological replicates. Error bars are the standard error of the mean. (TIF) [file pntd.0004364.s004.tif]

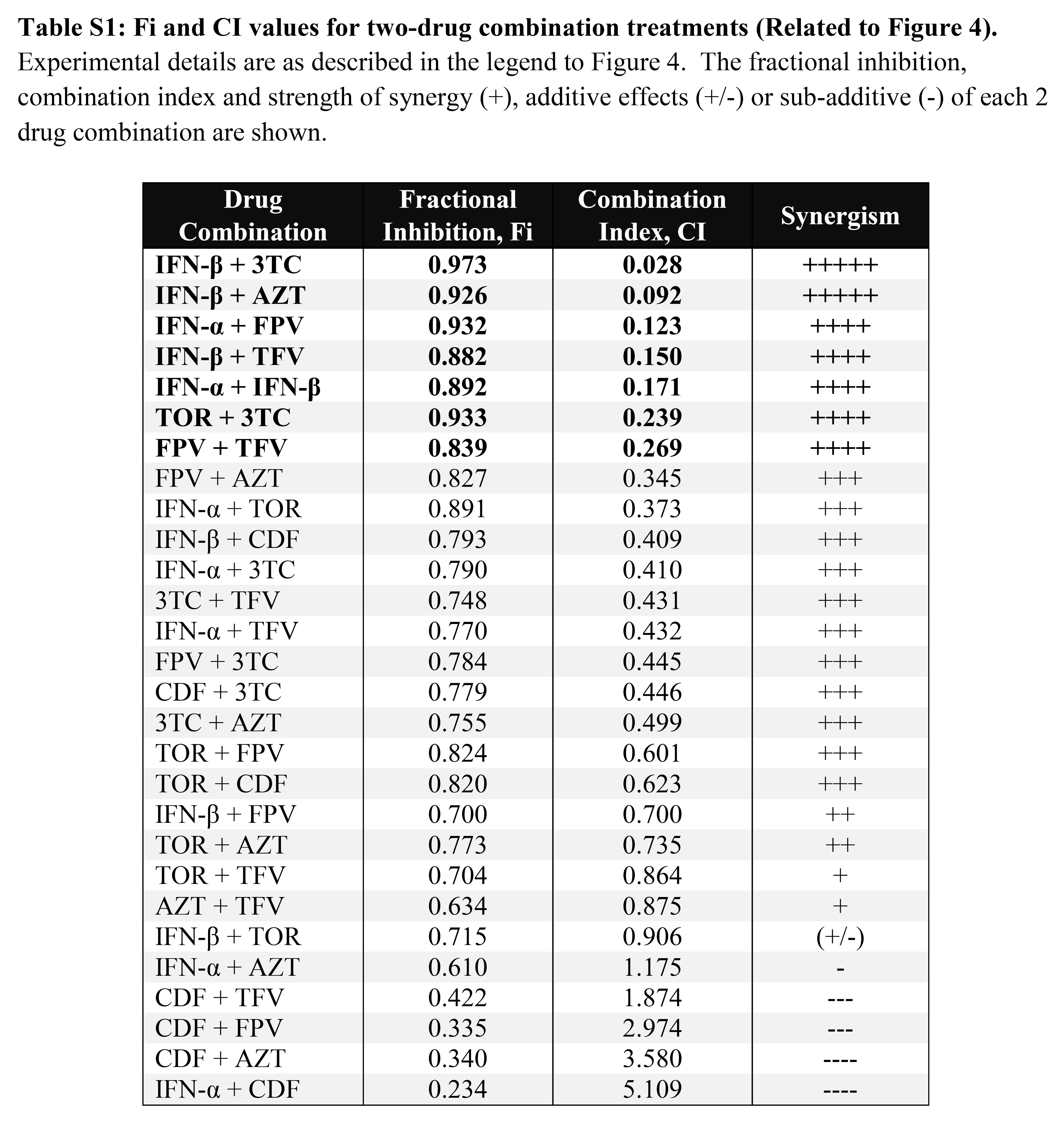

Supplement: S1 Table — Experimental details are as described in the legend to Fig 4. The fractional inhibition, combination index and strength of synergy (+), additive effects (+/-) or sub-additive (-) of each 2 drug combination therapy are shown. (TIF) [file pntd.0004364.s005.tif]

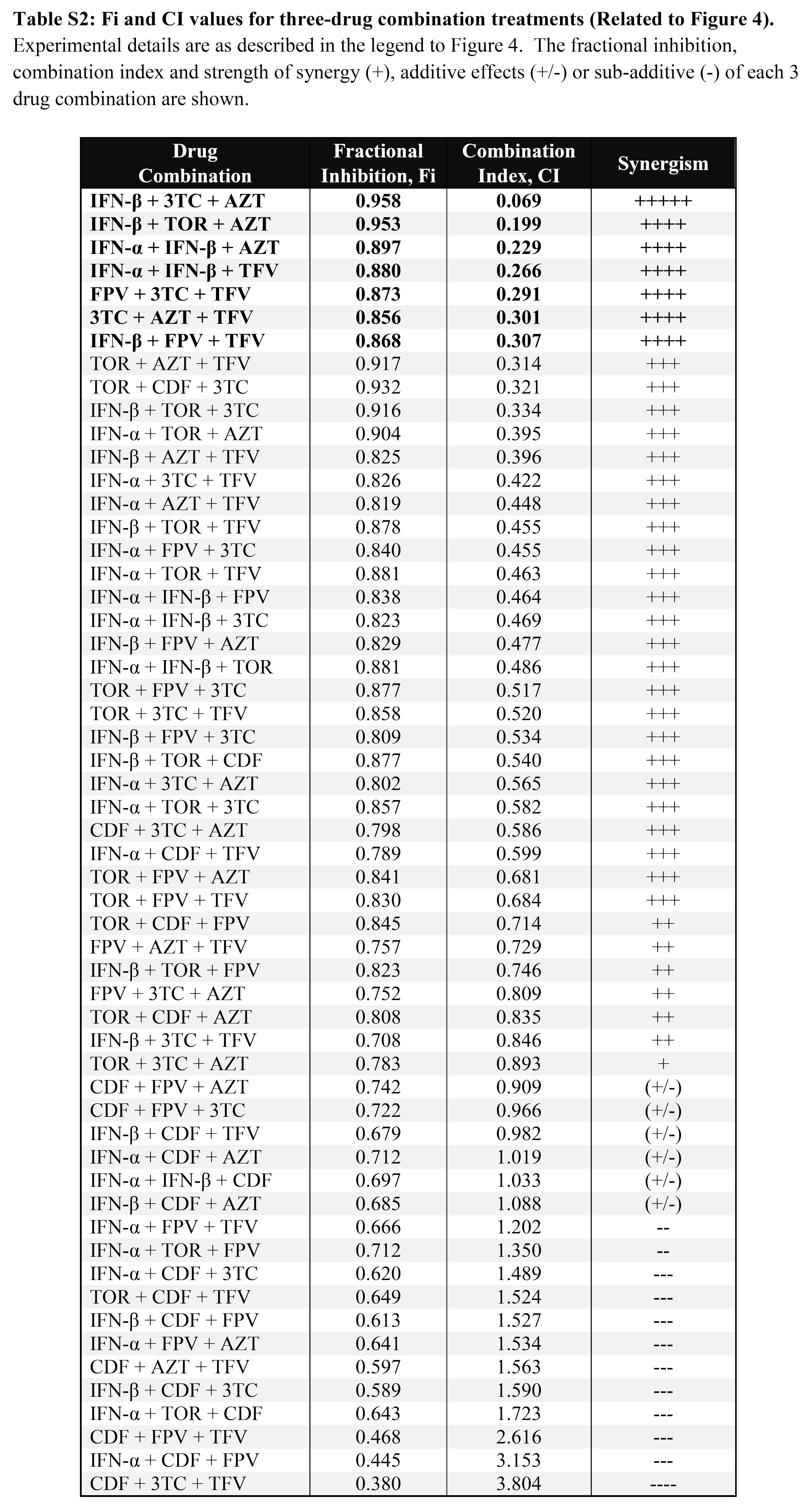

Supplement: S2 Table — Experimental details are as described in the legend to Fig 4. The fractional inhibition, combination index and strength of synergy (+), additive effects (+/-) or sub-additive (-) of each 3 drug combination therapy are shown. (TIF) [file pntd.0004364.s006.tif]
